# Special collection devoted to the VIII “metabolomics circle” conference organized by the Polish metabolomics society

**DOI:** 10.1007/s11306-024-02183-2

**Published:** 2024-10-13

**Authors:** I. Stanimirova, M. Daszykowski

**Affiliations:** Institute of Chemistry, University of Silesia in Katowice, Katowice, 40-006 Poland

Over the past decade, metabolomics has become a rapidly growing field that provides crucial insights into the biochemical processes underlying various physiological and pathological conditions. The changes or (bio-)chemical responses in the metabolite profile of a living organism that are observed due to disease, environmental factors, or genetic modifications can be identified, and the small molecules, which serve as intermediates and end products of metabolic pathways can then be quantified through various analytical techniques. Diverse chemometric methods are used to extract meaningful information and identify significant patterns or relationships within the complex chemical data collected from metabolomic studies.

This Special Collection (https://link.springer.com/collections/hceihdagea) includes several selected articles in metabolomics research that were presented during the VIII “Metabolomics Circle” conference organized by the Polish Metabolomics Society. The authors discussed very diverse problems and handled them using targeted and non-targeted metabolomics. A precise and sensitive methodology combining solid-phase microextraction, LC-MS/MS analysis and chemometrics was proposed to study the levels and distribution of four endocannabinoids in selected brain regions of female and male rats at different stages of development (Roszkowska et al.: https://link.springer.com/article/10.1007/s11306-023-02007-9). While endocannabinoids were studied as good indicators of the development and progression of central nervous system disorders, metabolites of *Lantana trifolia L*. leaves and fruits were screened for their antifungal and cytotoxic activity due to their use in the form of infusions and syrups to treat angina, coughs, and colds (Valerio et al.: https://link.springer.com/article/10.1007/s11306-023-02032-8). A new non-targeted cytotoxic biofluorescence assay was also developed on the basis of high-performance thin layer chromatography (HPTLC) and the methodology was tested for pink pepper (*Schinus* spp.) samples (Mügge et al.: https://link.springer.com/article/10.1007/s11306-023-02008-8). The proposed HPTLC method allowed for the direct detection of cytotoxic compounds on the adsorbent surface and presented a high selectivity to different substance classes in mid-polar and non-polar fruit extracts. The therapeutic efficiency of secondary leaves and bark metabolites of a new Egyptian hybrid, *Annona cherimola* × *Annona squamosa* was also studied (Mohammed et al.: https://link.springer.com/article/10.1007/s11306-022-01911-w). The authors examined extracts of different plant organs and identified their basic chemical components. In particular, the antioxidant activity of extracts, which were richest in alkaloids and polyphenolics, was determined and the most promising candidates were then screened for the in vivo treatment of gastric ulcer in rats. Tracing the changes in metabolite profiles of mice lung and plasma, including metabolites related to the GABA system as well as the urea cycle, citric acid cycle, and redox balance, helped in studying the pathogenesis of ventilator‑induced lung injury (Mao et al.: https://link.springer.com/article/10.1007/s11306-022-01914-7). Kvalheim et al. (https://link.springer.com/article/10.1007/s11306-022-01931-6) proposed an advanced multivariate regression approach for the assessment and adjustment of the influence of multicollinear and linear dependent covariates in order to study insulin resistance in 841 children by monitoring the comprehensive lipoprotein profiles described by proton nuclear magnetic resonance spectroscopy. The proposed projection approach made it possible to explain and interpret the influence of physical activity and adiposity using the collected data and to relate the observed changes to the lipoprotein features.

The articles in the Special Collection presented some key developments in metabolomics research such as innovative methodologies, therapeutic and biomedical insights, new assays, contributions to pathogenic studies, and advanced chemometric approaches for studying complex relationships. Together, they illustrated the growing scope of metabolomics research and its importance in the understanding of biochemical processes, with broad applications in exploring disease mechanisms, therapeutic discovery, and advanced analytical techniques. We hope you enjoy reading them.

